# On a New and Ready Mode of Producing Local Anæsthesia

**Published:** 1866-08

**Authors:** Benj. W. Richardson

**Affiliations:** Senior Physician to the Royal Infirmary for Diseases of the Chest


Local Anæsthesia.—An extract from the following article was copied into the May number of the Examiner, but the subject is of such importance that we have thought it best to republish the whole article.—Ed.

### ON A NEW AND READY MODE OF PRODUCING LOCAL ANÆSTHESIA.

By BENJ. W. RICHARDSON, M.A., M.D., F.R.C.P., Senior Physician to the Royal Infirmary for Diseases of the Chest.

Some years ago, I published in the columns of the *Medical Times and Gazette*, some researches for the production of local anæsthesia, by a process which I designated voltaic narcotism. Those researches, very much praised on the one hand, and very rudely and unfairly attacked on the other, failed in the end in leading me directly to any practical means of producing local insensibility applicable to surgical proceedings. The causes of failure were threefold. The apparatus required was very cumbersome; the application was painful; the result was uncertain. In the course of the past year a similar series of experiments have been made by an Italian physician; but whether in imitation of my previous labors or in ignorance of them, I do not know: they have proved equally unsatisfactory.

The researches on voltaic narcotism, although practically of little value, were not in reality without their use. Previously to making them I was quite conversant of the fact—indeed, I learnt it from Snow—that all the narcotics produced anæsthesia by the process of arresting oxidation; but I had still to learn what Snow himself had not reached, that arrest of oxidation meant, in the end, arrest of motion; and that anæsthesia, in truth, means the temporary death of a part influenced—*i.e.*, inertia in the molecules of the part.

Learning this, I discovered that voltaic narcotism had at its base a fault. My idea in it was, that by quickening the circulation of a part by galvanic stimulus, and by applying over the part where circulation was quickened a narcotic solution which the blood could absorb, I could so charge the blood locally with narcotic substance as to produce local insensibility. In feeble subjects, as the result proved, local narcotism could, in this way, be temporarily set up: but it was always attended with a certain amount of disorganization. In strong subjects it failed altogether, because such of the narcotic as might be absorbed was carried rapidly into the general circulation. In plain words, by the use of the galvanic current, I was committing the paradox of applying a form of motion for the indirect production of inertia.

The failures I experienced at the period referred to in no degree lessened my efforts to find a practical means for producing a local insensibility. They simply caused me to think more on the whole subject, and to invent new methods of inquiry. I came at length to the conclusion that Dr. James Arnott's plan of using extreme cold was the first true step of discovery, and that if it could be made easier of application, and at the same time could be combined with the use of narcotic fluid, an important advance in therapeutics would necessarily follow. For full four years this truth has been before my mind, and I have made numerous experiments with the view of demonstrating it. At one time I tried to freeze parts by the application of ice and salt, and then to inject by the hypodermic plan narcotic solutions into and beneath the frozen tissue. These experiments were never sufficiently satisfactory to allow of their publication. At last I hit upon a method which I am now about to describe, and which, although admitting of very considerable improvement, is sufficiently important to justify me in laying it before the profession.

THE ANÆSTHETIC SPRAY PRODUCER.

When the toy for diffusing eau de cologne in fine vapor over the skin, in the form of spray—which sometime ago found its way into our drawing-rooms—first came before me, it struck me at once that it might be applied to the production of local anæsthesia; and I set to work to try its applicability in this respect. I was soon afterwards assisted largely in my labors by taking advantage of Siegle's apparatus, with the hand-ball spray-producer invented by my valued friend Dr. Andrew Clarke, and supplied by the manufacturers, Messrs. Khrone & Seseman, of Whitechapel road.

With this apparatus I set myself to determine the degree of cold that could be produced by the vaporization of all the known volatile liquids, and I determined the fact that the intensity of the cold produced held a definite relationship to the boiling-point of the fluid used; the rule being that the lower the boiling-point the greater was the amount of cold exhibited. In these inquiries I employed a very delicate thermometer, directing the spray upon the bulb from half an inch to an inch and a-half from the point of the jet. By these means I learnt that with rectified sulphuric ether I could bring down the thermometer within 10 degrees Fahr. of zero, and that by directing the jet on the skin I could produce a certain definite and marked degree of local insensibility, but not sufficient for surgical purposes.

I next got Mr. Krohne to construct for me a hollow cylinder of thin metal, six inches long and three inches in diameter. In the circumference of this cylinder was a chamber one-eighth of an inch in diameter for containing ether. The ether communicated with a tube which was joined to an air-tube, as in Siegle's apparatus, and the centre of the cylinder was filled with ice and salt mixture. In this way the ether was reduced to zero, and when vaporized gave spray which brought down the thermometer six degrees below zero, and produced on the skin such entire insensibility that I could pass a needle through the part without sensation. On the 11th of December, 1865, I applied this process for the first time on the human subject for an operation. The patient was a lady, who required to have five front teeth extracted. I had previously administered chloroform to this lady for a tooth extraction, but the inhalation had produced so much irregularity in the action of the heart and other disagreeable symptoms, that I considered it inadvisable to repeat chloroform, and she herself was only too ready to give the local measure a trial. The extraction was performed by my friend Mr. Peter Matthews. On directing the ether spray first at a distance and then closely upon the gum, over the first central incisor on the left side, we observed, at the end of fifty seconds, that the gum had become as white as the tooth itself, and quite insensible. I then directed the vapor upon the tooth for twenty or thirty seconds more, and on the patient intimating that she did not feel, I suggested to Mr. Matthews to proceed. He extracted a very firm tooth without the slightest expression of pain. The process being continued in the same manner, he extracted three other teeth with the forceps. The other gave way, and had to be removed by the lever; but in all cases the result was equally good. Not a drop of blood was lost; there was no painful reaction; and the healing process proceeded perfectly. Our patient who was exceedingly intelligent, was specially requested to note every step of the operation, such as the applying of the forceps, the insertion of the blades beneath the gum, the loosening process, and the removal. She told us that in two of the extractions she felt nothing; that in one it seemed as though the jaw altogether were being pulled downwards, but without pain; that in another she was conscious of a kind of wrench or loosening but without pain, and that the introduction of the lever was attended with a momentary dull ache, just perceptible. On the whole, the process was quite as painless as when she took chloroform.

On December 13th, I applied the local anæsthetic to the same lady for the further extraction of nine teeth, Mr. Peter Matthews again operating. The results were equally good with the first seven, at which point, unfortunately, the apparatus partly ceased play. At the eighth tooth pain was felt, and at the ninth, the apparatus being out of play, the operation caused great pain. We regretted this much, although it gave us the information of the perfect action of the process when no mechanical obstacle interfered with it. The reason why the apparatus stopped play was very singular, and could hardly have been foreseen. It arose from the condensation of water derived from air in the air-tube, and from blocking up the fine jet with a little portion of ice.

In the next step of research, I got Mr. Krohne to make for me an apparatus with two spiral tubes, one the air-tube, the other a tube for ether; and I immersed these spirals in a closed chamber filled with ice and salt. The degree of anæsthesia at first produced was most intense, and Mr. Spencer Wells was good enough to allow me the opportunity of applying the process in a case where an operation was required for closing a perineal rupture. Unhappily the apparatus, from the very same cause as before, ceased to yield a current; water condensed and became frozen in the air-tube. The apparatus itself was also found too cumbersome for practical purposes; I therefore, in this trial failed to obtain any result.

By this time I had been led, very reluctantly, to the fact that the use of ice and salt for reducing the ether was a failure when the plan came to be tried in practice, nor could I see any ready way of preventing the difficulties that were brought before me. Added to these difficulties there was another, which has always attended my friend Dr. Arnott's plan, viz., that of getting the ice and salt readily for operation. To succeed, therefore, it was requisite to dispense with the ice and salt together.

In considering how this object could be achieved, it occurred to me that if a larger body of ether than is supplied by Siegle's apparatus could be brought through the same jet, by mechanical force, in the same interval of time, and with the same volume of air, a proportionate increase of cold must necessarily be produced. The theory was one of pure physics, admitting even of arithmetical demonstration, and running parallel with the lessons which had been taught me with respect to the cold produced by liquids having different degrees of boiling-point. The theory was put to the test at once, and proved correct to the letter. By driving over the ether under atmospheric pressure, instead of trusting simply to capillary action—or to suction, as in Siegle's apparatus—the spray evolved brought the thermometer within thirty seconds to four degrees below zero—the result that was desired.

Ascertaining this truth, I instructed Messrs Krohne & Sesemann to construct a proper apparatus. It consists simply of a graduated bottle for holding ether; through a perforated cork a double tube is inserted, one extremity of the inner part of which goes to the bottom of the bottle. Above the cork a little tube, connected with a hand bellows, pierces the outer part of the double tube, and communicates by means of the outer part, by a small aperture, with the interior of the bottle. The inner tube for d elivering the ether runs upwards nearly to the extremity of the outer tube. Now, when the bellows are worked, a double current of air is produced, one current descending and pressing upon the ether forcing it along the inner tube, and the other ascending through the outer tube and playing upon the column of ether as it escapes through the fine jet. By having a series of jets to fit on the lower part of the inner tube, the volume of ether can be moderated at pleasure; and by having a double tube for the admission of air, and two pairs of hand bellows, the volume of ether and of air can be equally increased with pleasure, and with the production of a degree of cold six below zero.

"By this simple apparatus, at any temperature of the day and at any season, the surgeon has thus in his hands a means for producing cold even six degrees below zero; and by directing the spray upon a half-inch test-tube containing water he can produce a column of ice in two minutes at most. Further, by this modification of Siegle's apparatus he can distribute fluids in the form of spray into any of the cavities of the body—into the bladder, for instance, by means of a spray catheter, or into the uterus by an uterine spray catheter.

"When the ether spray thus produced is directed upon the outer skin, the skin is rendered insensible within a minute; but the effects do not end here. So soon as the skin is divided the ether begins to exert on the nervous filaments the double action of cold and etherization; so that the narcotism can be extended deeply to any desired extent. Pure rectified ether used in this manner is entirely negative; it causes no irritation, and may be applied to a deep wound, as I shall show, without any danger. I have applied it to the mucous membrane of my own eye, after first chilling the ball with the lid closed.

"I have now employed this mode of producing local anæsthesia in four cases on the human subject. The first case was the extraction of a tooth from a lady, the operation being performed by my friend and neighbor Dr. Sedgwick, on January 24th of this year. On the 29th of the same month, I used it again on the same lady for the extraction of three very difficult teeth, Dr. Sedgwick again operating. The results were as satisfactory as in the previous case, where the ice and salt ether apparatus was used.

"I have used the apparatus also in connection with my friend Mr. Adams, who had a case at the Great Northern Hospital of deep dissecting abscess in the thigh of a young woman. In the abscess there was a small opening, which just admitted the director. I first narcotized around this opening, and the director being introduced, Mr. Adams carried his bistoury nearly an inch deep and one inch in the line of the director. I then narcotized the deep-seated parts, and enabled him to cut for another inch and a-half in the same direction. The director was then placed in the upper line of the abscess, the process was repeated, and the incision was carried two and a-half inches in that direction. The patient was entirely unconscious of pain, and after narcotising the whole of the deep surface, Mr. Adams inserted his fingers and cleared out the wound without creating the slightest evidence of pain.

"Afterwards, in the case of a lacerated wound, six inches long, in the arm of a boy, who had been injured with machinery, I narcotized while six sutures were introduced by Mr. Adams. The first needle was carried through without the anæsthetic, and caused expression of acute pain; the remaining eleven needles, after a few seconds' administration of the ether spray, were passed through painlessly. The twisting of the wire sutures gave no pain.

"These results are so interesting that I make no apology for bringing them at once before my medical brethren. I wish it to be distinctly understood that at the present moment I only introduce the method here described for the production of superficial local anæsthesia. It is, I believe, applicable to a large number of minor operations, for which the more dangerous agent chloroform is now commonly employed—I mean such operations as tooth extraction, tying nævus, tying piles, incising carbuncles, opening abscesses, putting in sutures, removing small tumors, removing the toe-nail, dividing tendons, operating for fistula, removing cancer of the lip, and other similar minor operations which I need not mention. The process may also be applied to reduce local inflammation.

"In course of time, and guided by experience and the advancement of science, we may, however, expect more. If an anæsthetic fluid of negative qualities, as regards irritation of nerve, and which has a boiling point of 75° or 80° can be obtained from the hydrocarbon series, the deepest anæsthesia may be produced, and even a limb may be amputated by this method. It may also turn out that certain anæsthetics may be added to the ethereal solution with advantage, such as small quanties of chloroform, or some of the narcotic alkaloids, if they could be made soluble in ether. A solution of morphia and atropia combined, if they could be diffused through ether, which at present seems impossible, could thus be brought into action so as to cause deep insensibility. In operating on the extremities it would be good practice to stop the current of warm blood by making pressure above on the main artery.

"Reaction from the anæsthesia is in no degree painful, and hemorrhage is almost entirely controlled during the anæsthesia.

"One or two precautions are necessary. It is essential, in the first place, to use pure rectified ether; methylated ether causes irritation, and chloroform, unless largely diluted with ether—say one part in eight—does the same.

The *modus operandi* of this process is exceedingly simple. It acts at first merely by extracting force, and afterwards, when the nervous filaments are exposed, by preventing the conveyance of force through them. To be plain, sensation means the conveyance of force or motion from the extreme parts to the brain. The motion is communicated by the blood in the form of heat: it is communicated to the nervous filaments, and by them is conveyed to the sensorium. This is passive sensibility. When we irritate a nervous fibre, as by a cut, we communicate more motion rapidly along that fibre and cause pain. This is active or exalted sensibility. To remove sensibility, therefore, we must adopt one of three processes: we must remove or render inert the sensorium; we must stop the evolution of force generally by arresting oxidation of blood; or we must rob the body locally of its force beyond that with which it is constantly being renewed. We see the first of these processes in action in cases of pressure on the brain, as from injury or effusion of blood; we see the second whenever we produce general anæsthesia by charging the blood we chloroform or other analogous anæsthetic; and we see the third when, by means of extreme cold, we rob the local part of the force that has been brought to it by the blood.

The problem of local anæsthesia will consequently be quite solved when by a rapid process we can exhaust the natural force of a part as fast as such force is evolved in the local structure; and especially when with this we can combine the action of a substance which for the moment controls, as by compression, the conducting power of nerve matter. These two latter objects are to a large extent carried out by the method I have described above.—*London Medical Times and Gazette*, Feb. 3, 1866.

